# The Effects of Age and Cytomegalovirus on Markers of Inflammation and Lymphocyte Populations in Captive Baboons

**DOI:** 10.1371/journal.pone.0107167

**Published:** 2014-09-22

**Authors:** Erin L. Willis, Richard Eberle, Roman F. Wolf, Gary L. White, Dianne McFarlane

**Affiliations:** 1 Department of Physiological Sciences, Center for Veterinary Health Sciences, Oklahoma State University, Stillwater, Oklahoma, United States of America; 2 Department of Veterinary Pathobiology, Center for Veterinary Health Sciences, Oklahoma State University, Stillwater, Oklahoma, United States of America; 3 Department of Comparative Medicine, University of Oklahoma Health Sciences Center, Oklahoma City, Oklahoma, United States of America; City University of New York (CUNY), United States of America

## Abstract

The human immune system undergoes age-related changes that can lead to increased disease susceptibility. Using the baboon as a model for human immune system aging, we examined age-related changes in relative and absolute numbers of T cell subpopulations, cytomegalovirus (CMV) titer and markers of inflammation. In addition, the effect of gender, social status and peer group on lymphocyte subpopulations was determined. Relative and absolute numbers of total lymphocytes (CD3+), T helper cells (CD4+), and cytotoxic T cells (CD8+) increased with age. The proportion of naïve T cells (CD45RA+) decreased, while the total number of cells negative for the co-stimulatory receptor, CD28 (CD28-) increased in an age-dependent manner. Furthermore, CMV titers were negatively correlated with the number of naive CD4+ cells. IL-6 and cortisol concentration were also negatively associated with T cell subpopulations. Additionally, socially dominant baboons exhibited decreases in naïve CD4+ and CD8+ cells (by 65% and 52%, respectively) compared to subordinate animals. These results suggest that factors such as CMV exposure and inflammation may contribute to the age-related decline in immune health and indicate that factors like social status should be considered when studying immunosenescence in animal models.

## Background

It is widely recognized that age negatively affects the immune system by causing dramatic changes on both a cellular and systemic level [1], [2]. The progressive weakening of cellular and molecular functions of the aging immune system is known as immunosenescence. Immunosenescence is accompanied by lymphocyte changes that include the accumulation of memory T cells, decreases in naïve lymphocytes and impaired effector T cell responses. In addition, a progression of chronic low-grade inflammation is observed during aging [3]. Elderly populations frequently suffer from an increase in disease and infections as the aged immune system responds inappropriately. Furthermore, older persons also exhibit reduced immune responses to vaccinations intended to prevent infections [4], [5]. A better understanding of age-associated immune changes and other factors that may contribute to immune dysfunction in the elderly will aid in the development of more appropriate preventative and therapeutic measures for many conditions that afflict aged populations.

The immune system functions through both innate and adaptive immune responses and lymphocytes that facilitate cell-mediated immune responses are essential in this. Although lymphocytes can be involved in both innate and adaptive responses, their main role is through adaptive immunity involving two main cell types, CD4 helper and CD8 cytotoxic T cells. Naïve T cells are populations of CD4 or CD8 cells that are yet to encounter an antigen and thus, are not yet activated [6], [7]. Lymphocytes are activated through a cascade of events, which typically involves the co-stimulatory receptor, CD28 [8]. In reaction to an immune stimulus, activated CD4 cells are responsible for mediating immune responses by the secretion of specific cytokines and by activating B lymphocytes, cytotoxic T cells and other non-immune cells. Activated, cytotoxic CD8 lymphocytes then destroy target cells infected with virus by inducing apoptosis. Once lymphocytes have been exposed to an antigen, they can be converted into memory T cells which are able to mount a quicker and stronger amnestic immune response to an antigen encountered previously [7], [9], [10].

As one ages, the population and functionality of these lymphocyte subsets can change, leading to dysfunction in cell-mediated immunity. It is thought that antigenic stress from immune-surveillance against chronic, latent viruses can contribute to the age-related immune dysfunction by promoting the loss of naïve cells and the accumulation of incompetent memory lymphocytes [11]. In particular, the chronic β-herpesvirus cytomegalovirus (CMV) is suspected to play a major role in contributing to immunosenescence and in reducing the ability of the elderly to appropriately respond to an immune stimulus [11]. Furthermore, there is also evidence that other factors, such as gender and social status, can contribute to immune health in humans and other animals [12]–[14].

Although studies in people have shown a link between chronic pathogens and immunosenescence, the corresponding cause and effect relationship remains elusive. Baboons are an excellent animal model for studying the immune system. They share a similar susceptibility to pathogens with humans and are commonly infected with chronic herpesviruses that are closely related to the homologous herpesviruses of humans, including CMV. Previous studies by our laboratory revealed that baboons show age-related immune changes typical of those found in people and other species [15], [16]. More specifically, we found that age was positively correlated with the markers of inflammation, C-reactive protein (CRP) and the pro-inflammatory cytokine, interleukin-6 (IL-6) and that serum cortisol was negatively impacted by age [15]. Moreover, a study by Jayashankar et al. examined the effect of age on lymphocyte populations in baboons [17]. They found an age-related increase in the relative proportion of lymphocytes (CD3+), and an increase in the relative number of T helper CD4 cells (CD4+) and cytotoxic CD8 lymphocytes (CD8+). They also found that the proportion of lymphocytes expressing the co-stimulatory receptor CD28, which is required for activation of CD4 and CD8 cells, did not change [17]. However, this study did not report on the absolute age-related changes in lymphocyte subsets and did not evaluate naïve lymphocytes. Furthermore, they did not examine the potential relationship of CMV, markers of inflammation, or of other physiological and non-physiological factors with changes in lymphocyte populations.

Therefore, we sought to more extensively define age-related changes in lymphocyte subsets as well as examine the relationship between other factors (such as CMV antibody titers, markers of inflammation and social status) and age-related changes in the immune system of adult baboons. The specific goals of this study were to determine the effect of age on the relative and absolute numbers of different lymphocyte populations, including those expressing the co-stimulatory receptor CD28 and the naïve cell marker CD45RA+, and to examine the possible correlation of CMV IgG titers, markers of inflammation, and other physiological/non-physiological factors with these changes.

## Methods

### Animals and sample collection

The population analysis was completed on normal adult baboons housed in hierarchical peer groups of approximately 60 to 80 animals at the National Baboon Research Resource, Department of Comparative Medicine, University of Oklahoma Health Sciences Center (OUHSC). The baboons resided in the OUHSC baboon breeding colony under the daily care of three clinical veterinarians. Male and female baboons were group-housed in large outdoor corrals with attached indoor group cages. All housing was equipped with enrichment items for climbing, exercise and play. They were fed a commercial diet of monkey chow along with fresh fruits and vegetables. Water was provided ad libitum. Blood samples were collected on-site during routine semiannual health checks to avoid the need for additional procedures that could lead to discomfort and stress. Venous blood was collected (in the morning) under anesthesia (intramuscular ketamine, 10 mg/kg) from all adult baboons over 6 years of age included in the health assessment. No pregnant animals were anesthetized and no animals were euthanized for the study. Whole blood was collected into vacutainer tubes containing EDTA as an anticoagulant for same day analysis by flow cytometry. An additional sample was collected and clotted before being centrifuged at 500 g for 15 min for serum collection. Serum was transported to the laboratory on ice and frozen at −80°C until analysis. Blood was collected from 33 females and 5 males ranging in age from 6 to 26 years old with a mean age of 14 years. It is estimated that this baboon age range would correspond to a human age range of approximately 18–78 years old [18], [19]. The average lifespan of baboons in captivity is approximately 21 years of age [20] and our population included 14 young adult (<13 years), 14 middle aged (13–17 years), and 10 aged baboons (>17 years). Animal gender along with non-physiological factors such as social status (graded into dominant, intermediate, or subordinate social group status) and peer group were recorded. *Ethics Statement*: All animal procedures were approved by the University of Oklahoma's Institutional Animal Care and Use Committee (permit number 12–160).

### Lymphocyte numbers and Flow Cytometry

Total lymphocyte numbers were determined from a complete blood cell count and blood differential via an automated hematology analyzer (scil Vet abc, Gurnee, IL) and Wright Geimsa-stained blood smears. Flow cytometry was used to determine relative lymphocyte subset percentages; absolute cell subset numbers were then calculated. Samples were analyzed for CD3+ (total) lymphocytes, CD4+ (helper) lymphocytes, CD8+ (cytotoxic) lymphocytes, CD4+ CD45RA+ naïve helper lymphocytes, CD8+ CD45RA+ naïve cytotoxic lymphocytes, CD4+ CD28- lymphocytes and CD8+ CD28- lymphocytes using flow cytometry. Whole blood (100 µl) was stained with the appropriate antibodies for 20 min in the dark at room temperature. Antibodies used were CD3-allophycocyanin (APC; clone SP34-2, Becton-Dickinson, San Jose, CA), CD4-fluorescein isothiocyanate (FITC; clone L200, Becton-Dickinson), CD8 phycoerythrin-Cy7 (PE-Cy7; clone RPA-T8, Becton-Dickinson), CD45RA-APC (clone MEM-56, Invitrogen, Life Technologies, Carlsbad, CA) and CD28- PE (clone CD28.2, Becton-Dickinson). Samples were then treated with erythrocyte lysis buffer (containing 0.17 M NH_4_Cl, 0.1 M KHCO_3_ and 0.9 mM tetrasodium EDTA), allowed to incubate for 10 min in the dark at room temperature, then centrifuged at 600 g for 5 min. Pelleted cells were collected and washed twice with Hanks buffer containing 0.2% BSA. Cells were then fixed with cold BD Cytofix (Becton-Dickinson) according to the manufacturer's protocol and re-suspended in 500 µl of cold PBS containing 0.2% BSA and 0.1% NaN_3_. Samples were stored on ice until analysis with a FACSCalibur flow cytometer (Becton-Dickinson). Unstained cells, an isotype control and single-stained controls for each antibody were used to define the position of the negative cells and set the spectral compensation for multicolor experiments. Lymphocyte subsets were then determined using a CD3 gating strategy. Data were expressed as numbers of cells per cubic millimeter of blood (absolute numbers) or percent positive lymphocytes (relative numbers).

### Enzyme-linked immunosorbent assays for baboon cytomegalovirus, interleukin-6, cortisol, C-reactive protein and serum amyloid A

A standard indirect enzyme-linked immunosorbent assay (ELISA) for baboon CMV, previously developed and described elsewhere for use in this species [21], was used to measure CMV antibody titer in baboon sera. Baboon CMV-positive control sera obtained from OUHSC colony baboons shedding infectious CMV and CMV negative control sera obtained from OUHSC specific-pathogen-free baboons were used as controls. Results were expressed as OD_490_.

Inflammatory state was assessed by evaluating serum concentration of pro-inflammatory biomarkers including IL-6, CRP and serum amyloid A (SAA) as well as the anti-inflammatory hormone cortisol. IL-6 was measured using a non-human primate–specific commercial ELISA (U-Cytech, Utrecht) validated for use in baboons (assay lower limit of detection of 1 pg/ml) [15]. The concentration of CRP was determined using a high sensitivity human-specific ELISA kit (MP Biomedicals, Orangeburg, NY), validated previously for use in baboons by our laboratory (assay lower detection limit of 0.1 mg/L) [15]. The acute phase protein SAA was determined using a multispecies Phase SAA ELISA kit (Tridelta Development Limited, Maynooth, County Kildare, Ireland). Total serum cortisol was measured by a human-specific ELISA (Neogen Corporation, Lexington, KY; assay lower detection limit of 0.04 ng/ml). All assays were completed according to the manufacturers' directions. However, because standard SAA concentrations have not been published for baboons, SAA concentrations were not calculated from the standard curve. Instead, SAA was expressed as OD_450_, similar to the method for determination of SAA in rhesus macaques, described by MacGuire et al. [22].

The SAA and cortisol assays were validated for use in baboons by demonstrating parallelism between diluted pooled baboon serum samples to a standard curve, percent recovery and linearity of diluted baboon serum samples spiked with known concentrations of substrate. All samples were analyzed on a single ELISA plate per assay and the intra-assay coefficients of variance for the control samples were below 10% for each assay.

### Statistical Analyses

Table 1 includes details of the descriptive statistics for study animals. Data were tested for normality and homogeneity of variance prior to statistical analyses and were log-transformed, as needed. To determine the effects of age, CMV antibody titers, markers of inflammation, gender, social status, and peer group on lymphocyte subsets, backward stepwise regression was performed. Multiple linear regression was then performed with significant factors (relative CD4+ and CD4+ CD45RA+ and absolute CD3+ and CD4+) or when age was the only factor retained in the model (all other T cell populations) simple linear regression was performed. Pearson's correlation coefficient was used to measure of the degree of linear dependence between lymphocyte subsets, CMV antibody titer load, IL-6, cortisol, CRP, and SAA. T-tests were used to determine differences between pairs of means in the subordinate versus high social status baboons. Statistical significance was defined as *P*<0.5. JMP 7 Statistical Discovery (SAS, Cary, NC) was used for all statistical analyses.

**Table 1:** Distribution statistics of baboons by study.

			Animal Number (n)	
			Relative T cells	Absolute T cells
		AGE		
**A. Animal Distribution for effects of age on lymphocytes**	Young-adult	>13 years	14	12
	Middle-aged	13–17 years	14	12
	Aged	<17 years	10	9
			38 Total;	33 Total;
			(33 Female; 5 Male)	(28 Female; 5 Male)
	Peer Group	Group 1	9	8
		Group 2	8	8
		Group 3	12	11
		Group 4	9	6
**B. Relationship of other factors to lymphocytes**			34–35	28–30
**C. Social Status Distribution**	Total High Social Status		15	15
	Total Subordinate		20	15
	Female High Social Status		10 (serum cortisol)	
	Female Subordinate		20 (serum cortisol)	

The population analysis was completed on normal adult baboons housed in hierarchical peer groups at the National Baboon Research Resource, Department of Comparative Medicine, University of Oklahoma Health Sciences Center. Male and female baboons were group-housed in large outdoor corrals (with attached indoor group cages). All blood samples were collected during routine health checks.

## Results

### The effects of age on relative and absolute numbers of lymphocyte subsets in baboons

**Figure 1:**
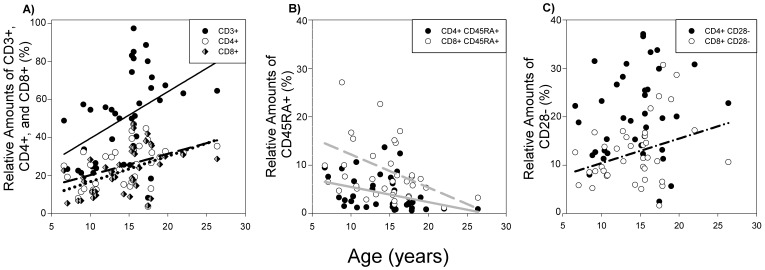
The impact of age on relative amounts of T cell subsets (n = 38). A) The effect of age on relative T cell populations. A) Relative number of CD3+, CD4+, and CD8+ increased with age (*P*<0.05). B) Relative number of naïve lymphocytes, CD4+ CD45RA+ and CD8+ CD45RA+, were negatively correlated with age (*P*<0.01). C) While age was positively correlated with the relative number of CD8+ CD28- lymphocytes (*P*<0.05), age did not impact relative amounts of CD4+ CD28- cells (*P* = 0.36). The black line represents the results of linear regression analysis for CD3+, the black dashed line for CD4+, the black dotted line for CD8+, the grey line for CD4+ CD45RA+, the grey dashed line for CD8+ CD45RA+, and the black dash-dotted line for CD8+ CD28- lymphocyte subtypes.

Consistent with previous studies [17], there was a significant positive impact of age on the relative proportion of CD3 lymphocytes (n = 38; Figure 1A; r = 0.41, *P* = 0.01). Likewise, when lymphocytes were further characterized as T helper CD4+ and cytotoxic CD8+ subsets, age also positively influenced the relative amounts of CD4+ and CD8+ lymphocytes (Figure 1A; r≥0.43, *P*<0.01). In contrast, the relative proportion of naïve T cell subsets (CD4+ CD45RA+ and CD8+ CD45RA+), both decreased with age (Figure 1B; r≤−0.35, *P*<0.05). The proportion of CD8+ lymphocytes negative for the co-stimulatory receptor CD28 also increased with age (Figure 1C; r = 0.32, *P*<0.05). However, there was no effect of age on relative levels of CD4+ CD28- cells (Figure 1C; not significant, *P* = 0.36).

**Figure 2:**
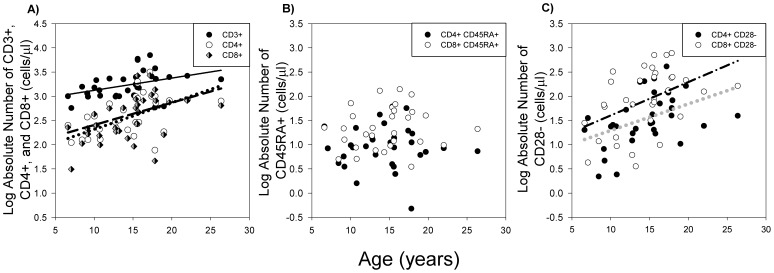
The impact of age on absolute cell numbers of T cell subsets (n = 33). A) Absolute number of CD3+, CD4+, CD8+ T cells increased in an age-dependent manner (*P*<0.05). B) The absolute number of CD45RA+ cells was not correlated with age (*P*≥0.46). C) Absolute numbers of cell negative for CD28 (CD28-) increased with age (*P*≤0.01). The black line represents the results of linear regression analysis for CD3+, the black dashed line for CD4+, the black dotted line for CD8+, the grey dotted line for CD4+ CD28-, and the black dash-dotted line for CD8+ CD28- lymphocyte subtypes.

To further examine age-related changes in lymphocyte subsets in baboons, absolute lymphocyte levels were also measured in 33 baboons using complete blood cell count and blood differential results. Consistent with the results for relative amounts, age was positively correlated to the absolute values of CD3+ (Figure 2A; r = 0.40, *P*<0.05), CD4+ (Figure 2A; r = 0.49, *P*<0.01) and CD8+ (Figure 2A; r = 0.49, *P*<0.01) lymphocytes. In contrast, although the relative proportion of naïve T cells decreased with age, a corresponding decrease in the absolute numbers of CD4+ CD45RA+ and CD8+ CD45RA+ cells was not observed (Figure 2B; not significant, *P*≥0.46). However, the absolute number of both CD4+ CD28- and CD8+ CD28- cells increased with age (Figure 2C; r≥0.44, *P*≤0.01).

### The impact of antibody titers for baboon cytomegalovirus as well as other physiological and non-physiological factors on immune health in baboons

**Figure 3:**
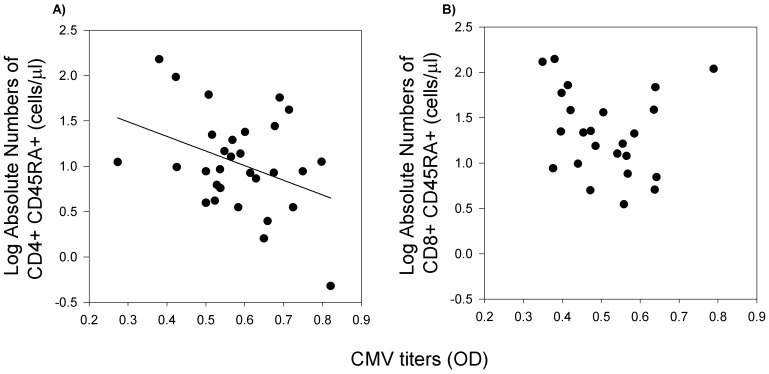
The correlation between CMV titer and absolute CD45RA+ subsets (n = 30). A) Correlation of absolute counts of CD4+ CD45RA+ naïve cells to CMV titer (r≤−0.33, *P*<0.05). B) Absolute counts of CD8+ CD45RA+ naïve cells were not associated with CMV titers (*P* = 0.53). The black line represents the results of linear regression analysis.

To test the hypothesis that CMV infection along with other physiological and non-physiological factors may impact immune health, we examined the relationship of CMV antibody titers, markers of inflammation, gender, social status, and peer group on T cell populations. High titers for CMV were associated with a decrease in relative proportion of CD4+ cells (data not shown; r = −0.33, *P*<0.05). Furthermore, absolute counts of CD4+ naïve cells (CD4+ CD45RA+) also decreased as CMV titers increased (Figure 3A; r = −0.37, *P*<0.05). Absolute counts of CD8+ naïve cells (CD8+ CD45RA+) were not correlated with CMV titers (Figure 3B; not significant, *P* = 0.53).

**Figure 4:**
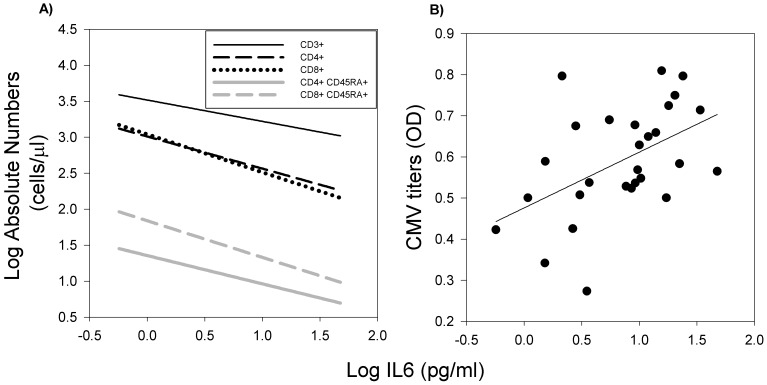
The relationship of serum IL-6 concentration to T cells and CMV titer (n = 28). A) Serum IL-6 was negatively associated with absolute counts of CD3+, CD4+, CD8+ lymphocytes and to naïve CD8+ CD45RA+ cells (r≥−0.46, *P*≤0.01). As IL-6 increased the number of naive CD4+ cells decreased, although not significantly (r = −0.35, *P* = 0.069). The black line represents the results of linear regression analysis for CD3+, the black dashed line for CD4+, the black dotted line for CD8+, the grey line for CD4+ CD45RA+ and grey dashed line for CD8+ CD45RA+ lymphocyte subtypes. B) Serum IL-6 was positively correlated with CMV titer (r = 0.49, *P*<0.01). The black line represents the results of linear regression analysis.

Unlike in the previous larger study [15], we did not find a significant effect of age on serum IL-6 in this smaller baboon study. However, as the concentration of IL-6 increased, absolute cell counts for multiple lymphocyte subpopulations decreased (Figure 4A). Serum IL-6 was negatively associated with absolute counts of CD3+, CD4+, CD8+ lymphocytes and to naïve CD8+ (*P*≤0.01) cells. Although the absolute numbers of naïve CD4+ cells also decreased as IL-6 increased, this failed to reach significance (r = −0.35, *P* = 0.069). Serum IL-6 concentration was also positively associated with CMV titer (Figure 4B; r = 0.49, *P*<0.01).

**Figure 5:**
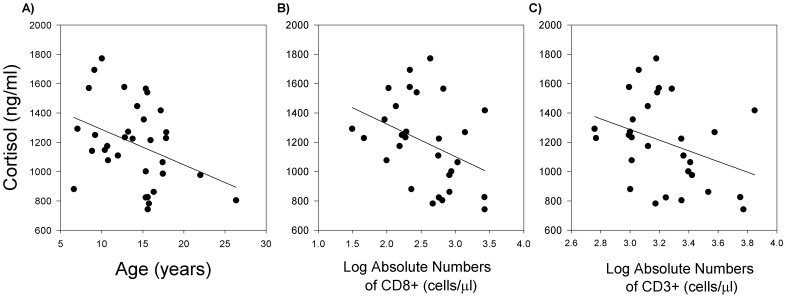
The relationship of total serum cortisol to age and lymphocyte subsets. A) Serum cortisol decreased in an age-dependent manner (n = 34; r = −0.37, *P*<0.05). B) Both relative (n = 34) and absolute counts (n = 30) of CD8+ decreased with increasing concentration of serum cortisol (r≤−0.34, *P*<0.05). Relative data not shown. C) A negative correlation was observed between cortisol concentration and absolute CD3+ cell count, although it failed to reach significance (n = 30; r = −0.34, *P* = 0.068). The black line represents the results of linear regression analysis for each graph.

Consistent with previous results [15], there was a negative effect of age on total cortisol concentration in serum (Figure 5A; r = −0.37, *P*<0.05). The concentration of cortisol was inversely related to both relative and absolute number (Figure 5B) of CD8+ cells (relative: r = −0.38, *P*<0.05; absolute: r = −0.34, *P*<0.05). There was also a negative impact of cortisol concentration on absolute counts of total CD3+ cells, although it failed to reach significance (Figure 5C; r = −0.34, *P* = 0.07). In contrast to the results for IL-6 and cortisol, there was not an association of the acute phase proteins CRP and SAA to T cells in this study, though these acute phase proteins were highly correlated to each other (data not shown; r = 0.70, *P*<0.0001).

**Figure 6:**
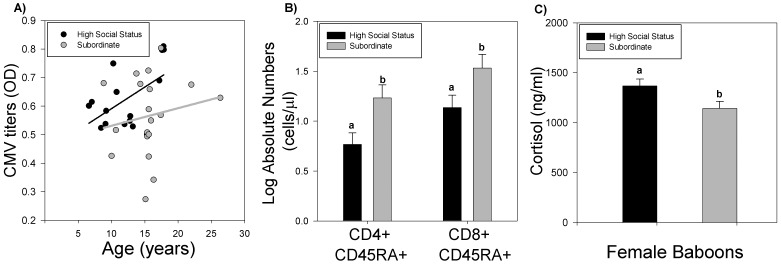
The impact of social status on immune health in baboons. A) High social status animals showed an age-dependent increase in CMV titers (n = 15), while CMV titers in subordinate animals were not affected by age (n = 20). The black line represents linear regression of the impact of age on CMV titers in high social status animals (r = 0.54; *P*<0.05), while the grey line represents linear regression in subordinate baboons (not significant). B) High social status animals also had a decrease number of CD4+ and CD+ 8 naïve cells compared to subordinate baboons (n = 15/group). C) Total serum cortisol concentration was increased in dominant female baboons (n = 10) compared to subordinate females (n = 20). Different lower case letters represent differences between groups, *P*<0.05.

Neither gender nor the specific peer group in which the animal lived was associated with changes in relative or absolute lymphocyte counts or CMV titers. However, social status had a considerable effect on different lymphocyte populations. When broken down into three social groups (dominant, intermediate, and subordinate), no difference was found between the dominant and intermediate baboons, and therefore these two groups were combined. However, when compared to subordinate animals, baboons of the combined higher social status group exhibited an age-dependent increase in CMV titers (Figure 5A; n = 16; r = 0.54; *P*<0.05) while there was no effect of age on titers in subordinate animals (Figure 6A; n = 19; r = 0.17, *P* = 0.48). High social status animals also showed greater age-related immune changes, as indicated by lower absolute counts of naïve lymphocytes (n = 15/group; Figure 6B). CD4+ naïve T cells were reduced by 65% (2.8-fold), while CD8+ naïve T cells were reduced by 52% (2.1-fold) in high social status baboons compared to subordinate animals. Additionally, a 20% (1.2-fold) higher concentration of serum cortisol was found among high social status females (n = 10) compared to subordinate females (n = 20; Figure 6C). All male baboons in the study population were dominant males and therefore, were excluded from analysis.

## Discussion

This study provides further knowledge of the normal aging process of the baboon immune system and examines other factors that could impact immune health in this species. It provides essential basic information necessary for developing a better understanding baboon aging, knowledge that is needed for the use of this species as a model for aging of the human immune system. The objective of this study was to confirm and expand on the previous findings of age-related change in T cell subset composition and inflammatory protein concentrations in baboons and to determine the interactions of CMV titer, as well as other physiological and non-physiological factors with these immune markers. Consistent with the results of Jayashankar et al. [17], the present study found that the relative values of the overall population of T cells (CD3+) as well as helper (CD4+) and cytotoxic (CD8+) T cells increased with age in our population. Age-related increases in the relative number of lymphocyte subset populations have also been found in primate species other than baboons, such as Chinese rhesus and cynomolgus macaques [23], [24]. The effects of age on lymphocytes were further characterized by examining the absolute cell counts in baboons, which were all shown to similarly increase with age.

Contrary to the increases in the numbers of total, T helper, and cytotoxic T cells, the relative number of naïve T- helper and cytotoxic T cells decreased with age. Naïve T cells help to provide protection against new and evolving pathogens. It is thought that decreases in the naïve T cell pool with age may cause an inadequate or inappropriate response to an immune stimulus [25], [26]. Interestingly, while the relative proportion of the naïve CD4+ and CD8+ T cells decreased with age, a parallel decrease in the absolute number was not observed, likely due to the increased total number of lymphocytes. It is important to note that the baboons used in this study were selected to investigate the development of age associated immune changes. Thus, the study included sufficient representation of young-adult (14 animals), middle-aged (14 animals) and aged animals (10 animals), but only comprised of three geriatric animals (≥20 years old). While the aged baboons (17.2–26 years old) were similar in age to old baboons used in other studies [19], [27], it is conceivable that additional changes may have been observed if more geriatric animals had been available to include on the present study. For example, it is possible that with the addition of more animals of extreme old age, a decrease in the absolute population of naïve T cells would be found since the most profound effects of age on the immune system are observed late in life [11], [28].

Studies in humans have found high frequencies of CD28- lymphocytes among the elderly [29], [30]. The present study found increases in the relative proportion of CD8+ CD28- lymphocytes, while the absolute numbers of both CD4+ and CD8+ CD28- lymphocytes increased with age in baboons, consistent with what has been reported in people. Activation of the CD28 co-stimulatory receptor promotes the differentiation of specialized lymphocytes [31]. People with high numbers of lymphocytes negative for CD28 show reduced responses to influenza vaccines as well as an increase in inflammatory diseases and inflammatory responses to immune stimuli [32]–[34]. While we did not observe an association between CD28- lymphocytes and the inflammatory markers examined in this study, we did not investigate CD28- lymphocytes activity in response to an immune stimulus. Consequently, the functional significance of the accumulation of CD28- cells in aged baboons warrants further investigation.

To explore factors that might contribute to the age-related changes in lymphocyte populations, we examined antibody titers for the chronic pathogen CMV as well as other physiological and non-physiological factors. Chronic infection with pathogens like CMV has been suggested to contribute to immunosenescence by causing the loss of naïve cells and increases in incompetent memory lymphocytes [11], [35], [36]. Cytomegalovirus is a chronic β-herpesvirus [37] and is a particularly significant potential contributor to immunosenescence due to its continual stimulation of the immune system. Cytomegalovirus seldom causes symptoms in healthy people, but the immune system is constantly responding to viral proteins that may impair immune effectiveness over time [38]. The present study found a negative association between CMV antibody titers and CD4+ and CD4+ CD45RA+ subtypes. Similarly, Olson et al. reported that lower CD4+ naive cells were strongly associated with titers for CMV in humans [39]. Although there is some evidence in humans that CMV-specific CD4+ T cells can be involved in some antiviral responses, anti-viral immune responses are typically mediated by CD8+ T cells [40], [41]. Future studies examining CMV viral load in addition to antibody titers will help to clarify the impact of CMV on age-related lymphocyte changes of both CD4+ and CD8+ subtypes.

High concentrations of cortisol and the pro-inflammatory cytokine IL-6 in serum were associated with reduced numbers of T cells. An increase in cortisol concentration was correlated with reduced numbers of cytotoxic T cells. This finding is consistent with reports in other species showing that treatment with the synthetic glucocorticoid dexamethasone reduces absolute cytotoxic CD8+ T cell numbers [42]. Similarly, the present study found that as the concentration of the cytokine IL-6 in serum increased, absolute counts of total T cells, T helper, cytotoxic and naïve lymphocytes decreased. High IL-6 was also positively associated with titers for baboon CMV in this study. In people, inflammation can promote immune system dysfunction and is considered a major risk factor for chronic aging diseases [43]-[46]. For example, in subclinical atherosclerosis patients, levels of IL-6 are correlated with decreases in naive CD4+ T cells [39]. Moreover, human CMV is able to induce expression and secretion of pro-inflammatory factors by human cells [47]. In contrast to what was observed with cortisol and IL-6, neither CRP nor SAA concentration was associated with T cell numbers. CRP and SAA are acute phase proteins released from the liver in response to inflammatory cytokine stimulation and largely activate innate, rather than adaptive, immunity [48]. Although there is evidence that they are associated with certain diseases in the elderly [49], [50], our results suggest that these acute phase proteins are not likely to be reliable surrogates for determining specific adaptive immune changes.

We did not observe lymphocyte differences between male and female baboons in the current study. However, since our population was a breeding group, it was skewed towards females. Nevertheless, we did find an effect of social status on certain T cell subsets in baboons and our results indicated that dominance could have some detrimental effects on the immune system. Previous studies in baboons and other non-human primate (NHP) species have found differences in health between high ranking and subordinate animals. Tung et al. found that in rhesus macaques, low-ranking animals had a reduced relative proportion of CD8+ T cells and tended to overexpress inflammation-related genes when compared to dominant animals. However, the study examined the effects of social status in experimentally constructed social groups [13]. The social status of our baboon population was not experimentally altered by introducing new animals or by regrouping of animals. Unlike the findings of Tung and colleagues, we did not find a relationship of social status to CD8+ cells in our baboon population. However, we did find that higher social status animals had an age dependent increase in CMV titers and an unexpected reduction in naïve T cells when compared to subordinate animals. Dominant female baboons also had higher concentrations of total cortisol in serum than subordinates. Similarly, Gesquiere et al. found that in wild savannah baboons, alpha males showed higher stress hormone levels compared to beta males, also suggesting that dominance might come at some costs of health [12].

## Conclusions

The current study provides a further understanding of the effect of age on the immune system of baboons. We found that both the relative and absolute numbers of helper and cytotoxic T cells increased with age in baboons. We also observed that aged baboons had proportionally less naïve T cells and an increase in the absolute number of T cells negative for the co-stimulatory marker CD28. Both of these changes occur in aged people and are associated with a loss of immune competency. Moreover, we found CMV antibody titers, IL-6, cortisol and social status were associated with changes in baboon lymphocyte subpopulations. These results suggest that baboon lymphocytes, in particular subsets important for functionality of the immune system later in life, age in a manner similar to what has been observed in people. The results also indicate that non-physiological factors, such as social status, can affect immune health and should be taken into consideration when using NHPs in immune studies.

## Supporting Information

File S1 **Text data file for Willis et al., The effects of age and cytomegalovirus on markers of inflammation and lymphocyte populations in captive baboons.** File S1 contains all lymphocyte, CMV, IL-6, Cortisol, SAA and CRP measurement S1 data used in the current manuscript.
(XLSX)
